# Identification of Genomic Regions and Candidate Genes Associated with Body Weight and Body Conformation Traits in Karachai Goats

**DOI:** 10.3390/genes13101773

**Published:** 2022-09-30

**Authors:** Ahmed A. Easa, Marina Selionova, Magomet Aibazov, Tatiana Mamontova, Alexander Sermyagin, Anna Belous, Alexandra Abdelmanova, Tatiana Deniskova, Natalia Zinovieva

**Affiliations:** 1 Timiryazev Agricultural Academy, Russian State Agrarian University-Moscow, Timiryazevskaya Street, 41, Moscow 127550, Russia; 2 Department of Animal and Poultry Production, Faculty of Agriculture, Damanhour University, Damanhour 22511, Egypt; 3 L K Ernst Federal Research Center for Animal Husbandry, Dubrovitsy 60, Podolsk Municipal District, Moscow 142132, Russia

**Keywords:** GWAS, SNP, QTL, candidate genes, Karachai goats

## Abstract

The objective of this study was to identify the SNPs and candidate genes related to body weight and seven body conformation traits at the age of 8 months in the Russian aboriginal Karachai goats (n = 269) by conducting genome-wide association studies (GWAS), using genotypes generated by Goat SNP BeadChip (Illumina Inc., USA). We identified 241 SNPs, which were significantly associated with the studied traits, including 47 genome-wide SNPs (*p* < 10^−5^) and 194 suggestive SNPs (*p* < 10^−4^), distributed among all goat autosomes except for autosome 23. Fifty-six SNPs were common for two and more traits (1 SNP for six traits, 2 SNPs for five traits, 12 SNPs for four traits, 20 SNPs for three traits, and 21 SNPs for two traits), while 185 SNPs were associated with single traits. Structural annotation within a window of 0.4 Mb (±0.2 Mb from causal SNPs) revealed 238 candidate genes. The largest number of candidate genes was identified at Chr13 (33 candidate genes for the five traits). The genes identified in our study were previously reported to be associated with growth-related traits in different livestock species. The most significant genes for body weight were *CRADD*, *HMGA2*, *MSRB3*, *MAX*, *HACL1* and *RAB15*, which regulate growth processes, body sizes, fat deposition, and average daily gains. Among them, the *HMGA2* gene is a well-known candidate for prenatal and early postnatal development, and the *MSRB3* gene is proposed as a candidate gene affecting the growth performance. *APOB*, *PTPRK*, *BCAR1*, *AOAH* and *ASAH1* genes associated with withers height, rump height and body length, are involved in various metabolic processes, including fatty acid metabolism and lipopolysaccharide catabolism. In addition, *WDR70*, *ZBTB24*, *ADIPOQ*, and *SORCS3* genes were linked to chest width. *KCNG4* was associated with rump height, body length and chest perimeter. The identified candidate genes can be proposed as molecular markers for growth trait selection for genetic improvement in Karachai goats.

## 1. Introduction

Goat (*Capra hircus*) farming is widespread in almost every country all over the world, due to their low prices and high quality of their products, and so it is attracting new farmers and investors [1]. The highest numbers of goats in the world are located in developing countries under extreme climate conditions and under traditional farming systems [2].

In Russia, there are about 2 million goats of 10 breeds of various productivity directions [3]. Of the total number of goats in Russia, 80% are concentrated in three federal regions: Caucasus (40%), South (25%), and Siberia (15.5%) [4]. Karachai goats (Supplementary Materials Figure S1) are one of the most interesting breeds for research due to their ability to survive in the harsh conditions of the Main Caucasian Range at an altitude of 500–1200 m above sea level, which contains a high percentage of pastures located on extremely steep slopes covered with rare mountainous plants [5]. A feature of feeding in Karachai goats is the large consumption of medicinal herbs, berries and fruits, which grow in abundance on the slopes of the foothills and mountain pastures of the Caucasus [6]. Karachai goats belong to dual-purpose breeds and are used for meat and milk production. The adult males and females reach body weights of 63.0 and 44.5 kg, respectively. The duration of the lactation period is about 140–200 days. The average milk yield is 250–350 kg milk, but the best females are able to produce 500–550 kg milk. The average of fat and protein percent in milk of Karachai goat is 5.5% and 4.5%, respectively, while in some individuals it can reach 11.0% and 6.5%, respectively. At the age of 8 months the animals reach their market weight (30–35 kg). At the same age the goats are selected for further breeding. The most developed females, which have reached the body weight of 34–35 kg at 8 months can be inseminated in the year of birth [6].

Since the sequencing of the goat genome in 2010, the status of goat genetics has completely changed [7]. The progress of genome sequencing and genotyping technologies has made genome-wide association studies (GWAS) possible for identifying single nucleotide polymorphisms (SNPs) associated with phenotypes of interest [8]. GWAS is widely used to identify genetic regions and candidate genes associated with quantitative traits [9,10,11]. The progress of Illumina Goat 50 K SNP BeadChip has a big chance to aid in the discovery the genomic regions under the selection effect [12]. Body weight is a very important trait for the success and progress of small ruminants farming due to the economic value of this trait. Moreover, body conformation traits play an important role in breed identification and classification, and body conformation traits also have a positive correlation with body weight [13]. The identification of genetic regions for establishing variances in body weight and body conformation traits is highly important for selection [14].

Several previous studies have been performed to identify genomic regions related to body weight and body conformation traits in goats. In meat goats, EPH Receptor A5 (*EPHA5*) is a candidate gene linked to body length in Punjab goats [13]. At the same time, stromal interaction molecule 1 (*STIM1*), myosin light chain kinase (*MYLK*), and cell adhesion molecule 2 (*CADM2*) are genes associated with body weight, body length, and body height in different meat breeds [15,16,17]. Furthermore, in a previous study, 53 significant SNPs and 42 candidate genes (including *PSTPIP2*, *CCL19*, *FGF9*, *SGCG*, *FIGN*, and *SIPA1L*) were found to be linked to body height, body length, cannon circumference, chest depth, chest width and heart girth in Dazu Black Goats [18]. In dairy goats, the paired-like homeodomain 2 (*PITX2*) gene was linked to the body height and body length of Guanzhong and Hainan black goats [19]. In the same trend, the PR/SET Domain 6 (*PRDM6*) gene was associated with several body conformation traits such as cannon circumference, chest depth and chest width in Murciano-Granadina goats [20]. In dual-purpose goats, the prolactin receptor (*PRLR*) gene was found to be associated with body length, body height, chest depth, heart girth and cannon circumference in Shaanbei white cashmere goats [21]. Lim domain-binding 2 (*LDB2*), and low-density lipoprotein receptor-related protein 1B (*LRP1B*) genes was found to be correlated with body weight in Inner Mongolia goats; in the same study, prominin 1 (*PROM1*), F-box and leucine-rich repeat protein 3 (*FBXL3*), and mitogen-activated protein kinase 3 (*MAPK3*) genes were identified as candidate genes related to body weight [22]. To identify the significant SNPs and candidate genes linked to the growth and body conformation traits of indigenous Karachai goats, we conducted the genome-wide association studies using Goat SNP BeadChip (Illumina Inc., San Diego, CA, USA) and phenotypic records for body weight and seven body measurements at the age of 8 months, when the animals are selected for further breeding.

## 2. Materials and Methods

### 2.1. Animals, Sampling and Genotyping

A total of 269 Karachai goats from six breeding herds, including Darinsk, Kyzyl Kala, Maysky, Piatigorsky, Storozhevaya, and Uchkulan, were randomly selected for our study. The ear tissue samples were collected by trained personnel under strict veterinary rules in accordance with the rules for conducting of laboratory research (tests) in the implementation of the veterinary control (supervision) approved by Council Decision Eurasian Economic Commission No 80 (10 November 2017). The DNA was extracted using the DNA-Extron reagent kit (JSC Sintol, Russia) according to the manufacturer’s protocol. The concentration of double-stranded DNA was determined on a Qubit device (Thermofisher, Waltham, MA, USA). The purity of DNA was estimated by the degree of absorption at a wavelength of 260 and 280 nm, using a NanoDrop 8000 micro-spectrophotometer (Thermo Fisher Scientific, DE). The DNA samples were genotyped using the Illumina Goat SNP BeadChip (Illumina Inc., San Diego, CA, USA) comprising of 53,347 SNPs. 

### 2.2. Quality Control of Data

Quality control and filtering of genotyping data for each SNP and each sample was performed using the PLINK 1.9 software package (http://zzz.bwh.harvard.edu/plink/ accessed on 1 August 2020) using the following filters (corresponding commands in the PLINK software are given in brackets): call-rate for all studied SNPs for an individual sample is not less than 90% (--mind); call-rate for each of the studied SNPs for all genotyped samples is not less than 90% (--geno); minor allele frequency (MAF) greater than 0.01 or 0.05 (--maf 0.01); and deviation of SNP genotypes from Hardy–Weinberg distribution in the totality of tested samples with a significance *p*-value < 10^−6^ (--hwe). In addition, the linkage disequilibrium of the studied SNPs (LD score) was assessed with R^2^ < 0.2 with a step of 50 kb (--indep-pairwise). A total of 269 animals and 47,647 SNPs were included in our final data set after quality control.

### 2.3. Principal Component Analysis

The studied population of Karachai goats originated from six breeding herds. To evaluate the population stratification, we performed principal component analysis (PCA). PLINK v1.9 software was used to perform PCA.

### 2.4. Phenotypic Traits

The records for the body weight (BW) and seven body conformation traits including withers height (WH), rump height (RH), body length (BL), chest perimeter (CP), chest width (CW), chest depth (CD) and rump width (RW) were collected at age 8 months. Elimination of environmental and permanent effects by the method of generalized linear models for fitting descriptive statistics to analyze the normal distribution of the studied animals’ features were carried out by STATISTICA 10 software:

$$y=HY_{i}+Sex_{k}+b_{1}Age+animal_{j}+e,$$

where: *y*—the corresponding GLM (general linear model) animal phenotype; *HY_i_*—fixed effect “herd-year” of animal birth (*i* = 1–10); *Sex_k_*—the fixed effect of the sex of the animal (*k*—male, female); *Age*—the regression effect of age in days at the time the animal was assessed; *b*_1_—the regression coefficient of the model; *animal_j_*—the fixed effect of individual (*j* = 1–269) weighed by covariance structure for genomic relationship matrix (GRM, *N*(0, *Gσ_g_*^2^)) built from the genomic information using VanRaden’s method [23]; *e*—residual effect of the model.

### 2.5. Genome-Wide Association Studies

To identify associations of SNP markers with the studied pure (direct) phenotypic traits, we used multiple linear regression analysis implemented in PLINK 1.90, preliminarily adjusted studied population according to its structure (--genome, --covar). After a quality check, a total of 53,347 SNPs were used in this analysis. To confirm the significant influence of SNPs and identify significant regions in the goat genome, the Bonferroni null hypothesis test was used: threshold *p* < 1.05 × 10^−6^; 0.05/47647 SNP. Data visualization was carried out in the qqman package using the R programming language [24]. 

### 2.6. Gene Analysis

The SNP positions used for GWAS, specified according to the AdaptMap genome assembly, were converted into the ARS1.2 genome assembly and used for gene identification using the Ensembl Genes release 103 database web resource [25]. Structural annotation was performed for genomic regions covering a window of ±0.2 Mb from the identified SNP. The genes were considered as candidate genes when they (or part of them) were localized within selected 0.4 Mb window. For functional annotation and gene ontology (GO) term enrichment analysis for identified structural candidate genes, we used the Database for Annotation, Visualization and Integrated Discovery (DAVID) v6.8 software [26]. Significant annotation clusters were selected using an enrichment score of more than 1 and a *p*-value < 0.05.

## 3. Results

### 3.1. Population Stratification

**Figure 1:**
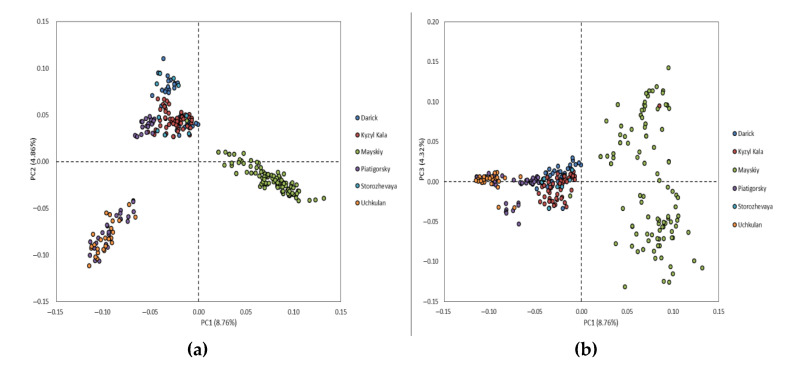
Population structure from the principal component analysis (PCA). PCA plots show the distribution of individuals of Karachai goats in the dimensions of two coordinates, i.e., (**a**) the first (PC1; *X*-axis) and second (PC2; *Y*-axis) principal components, (**b**) the first (PC1; *X*-axis) and third (PC3; *Y*-axis) principal components, with the percentage of total genetic variability, which can be explained by each of the two components, indicated within the parentheses; the individuals from the different herds are indicated by different colors.

The principal component analysis shows a distribution of the studied population between three clusters. The first principal component (PC1), which is responsible for 8.76% of genetic variability, clearly separated the Maysky herd from five remaining herds, while the principal component two (PC2), which explained 4.86% of genetic variability, distinguished the Darinsk, Kyzyl Kala and Storozhevaya herds from the Uchkulan herd. The individuals of the Piatigorsky herd were distributed between two clusters (Figure 1).

Considering the observed population stratification, we performed GWAS using the first two PCs as covariates.

### 3.2. Genome-Wide Association Studies

The descriptive GLM statistics for body weight and body conformation traits in Karachai goats at age 8 months are summarized in Table 1. The phenotypic value distribution by GLM model for measured traits in studied goats of the Karachai breed is shown in Supplementary Materials Figure S2. Descriptive statistics of the genomic inbreeding coefficient derived from the genomic relationship matrix in Karachai goats by herds revealed the low variation from 3.14 to 6.68% and had a normal distribution for all studied population (Supplementary Table S1, Supplementary Figures S3 and S4).

**Figure 2:**
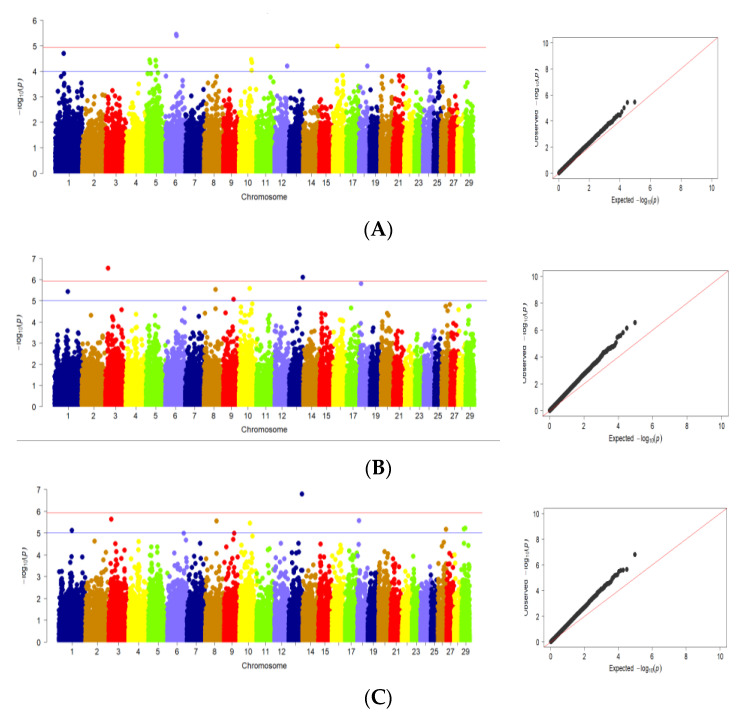

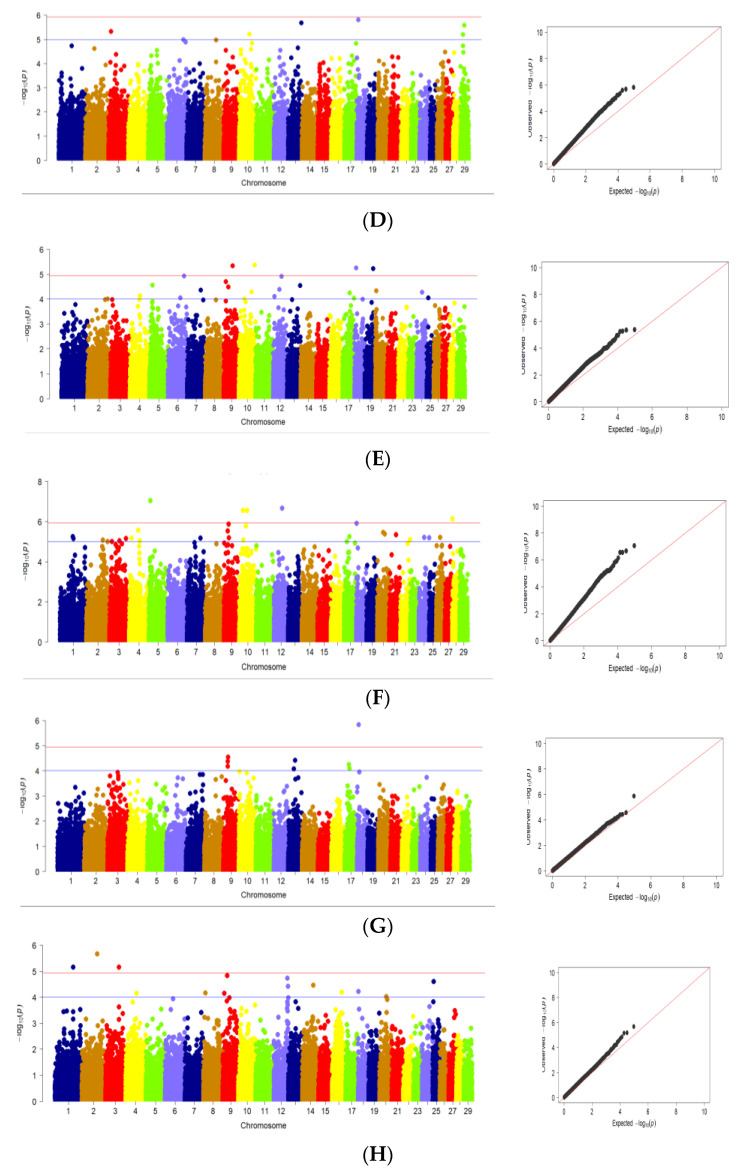
Manhattan plots and quantile–quantile (Q–Q) plots of GWAS for studied traits: (**A**) body weight; (**B**) withers height; (**C**) rump height; (**D**) body length; (**E**) chest perimeter; (**F**) chest width; (**G**) chest depth; (**H**) rump width. (Manhattan plots): distribution of single nucleotide mutations in goat chromosomes to the level of significance (−log10 (*p*) according to the suggestive probabilistic value (blue line, *p* < 0.0001) and the Bonferroni test (red line, *p* < 1.05 × 10^−6^) for the traits; (Q–Q plots): probability distribution quartile of expected and observed deviations from a normal distribution for confidence values. The dots are color-coded to visualize chromosome separation; the color does not carry any other semantic load.

The results of GWAS analysis for body weight and body conformation traits in Karachai goats at age 8 months are shown in Figure 2.

Using GWAS, significant SNPs associated with body weight at age of 8 months were found on Chr1, Chr5, Chr6, Chr10, Chr12, Chr16, Chr18 and Chr24 (Figure 2A). At the same time, significant SNPs related to withers height at age 8 months were located on Chr1, Chr3, Chr8, Chr9, Chr10, Chr13 and Chr18 (Figure 2B). For rump height, SNPs associated with this trait found on Chr1, Chr3, Chr8, Chr9, Chr10, Chr18, Chr26 and Chr29. This fact is due to the lower variability of these traits in the studied samples of goats (Figure 2C). For body length at age 8 months, significant SNPs were found on Chr3, Chr10, Chr13, Chr18 and Chr29 (Figure 2D). Significant SNPs associated with chest perimeter at age 8 months were located on Chr4, Chr5, Chr6, Chr7, Chr9, Chr10, Chr12, Chr13, Chr17, Chr18, Chr19, Chr20 and Chr24 (Figure 2E). Significant SNPs related to chest width were also found on Chr1, Chr2,Chr3, Chr4, Chr5, Chr7, Chr9, Chr10, Chr12, Chr17, Chr18, Chr20, Chr21, Chr22, Chr24, Chr26 and Chr28 (Figure 2F). On the other hand, SNPs significantly associated with chest depth at age 8 months were found on Chr9, Chr13, Chr17 and Chr18 (Figure 2G). Significant SNPs related to rump width were located on Chr1, Chr2, Chr3, Chr4, Chr8, Chr9, Chr12, Chr14, Chr16, Chr18 and Chr25 (Figure 2H).

Analysis of Q–Q plots (Figure 2A–H) shows that, for most traits, the observed *p*-values from the GWAS did not deviate significantly from the expected values, suggesting that the models for GWAS were reasonable. Greater deviations obtained at Q–Q plots for several traits including withers height, rump height, body length, chest width may be the result of some deviation from normal distribution for these traits (Supplementary Materials Figure S2). For this reason, the results produced for these traits should be treated with caution.

### 3.3. Identification of Significant SNPs

GWAS summary statistics are presented in Supplementary Materials, Table S2, and are summarized in Table 2.

GWAS for body weight showed 27 SNPs, including 5 genome-wide SNPs (*p* < 10^−5^) localized on Chr5, Chr6, Chr10 and Chr16, and 22 suggestive SNPs (*p* < 10^−4^) found on Chr1, Chr5, Chr10, Chr16, Chr18, Chr20, Chr24, Chr25 and Chr26. The greatest number of SNPs (6 SNPs, including 1 genome-wide SNP) was detected on Chr10 and Chr16. Part of the SNPs were localized as blocks of several neighboring SNPs, in particular, 2 SNPs (including 2 genome-wide SNPs) in the region of 60.5–61.8 Mb on Chr6, 3 SNPs (including 1 genome-wide SNPs) in the region of 56.4–59.5 Mb on Chr16, and 3 SNPs (including 1 genome-wide SNP) in the region of 70.9–73.3.4 Mb on Chr10. The greatest number of SNPs was found on Chr5 (6 SNPs, including 1 genome-wide SNP), Chr10 and Chr16 (4 SNPs, including 1 genome-wide SNP). GWAS for withers height trait revealed 49 SNPs, including 7 genome-wide SNPs (*p* < 10^−5^) found on Chr1, Chr3, Chr8, Chr9, Chr10, Chr13 and Chr18, and 42 suggestive SNPs (*p* < 10^−4^) localized on Chr2, Chr3, Chr4, Chr5, Chr6, Chr7, Chr8, Chr9, Chr10, Chr11, Chr13, Chr15, Chr16, Chr17, Chr20, Chr26 and Chr29. The greatest number of SNPs was found on Chr10 (9 SNPs) and Chr3 (5 SNPs, including 1 genome-wide SNP). GWAS for rump height showed 55 SNPs, including 9 genome-wide SNPs (*p* < 10^−5^) located on Chr1, Chr3, Chr8, Chr9, Chr10, Chr13, Chr18, Chr26 and Chr29, and 46 suggestive SNPs (*p* < 10^−4^) distributed among 20 autosomes, including Chr2, Chr3, Chr4, Chr5, Chr6, Chr7, Chr8, Chr9, Chr10, Chr11, Chr12, Chr13, Chr15, Chr16, Chr17, Chr18, Chr20, Chr26, Chr27 and Chr29. The greatest number of SNPs (6 SNPs, including 1 genome-wide SNP) was detected on Chr10, and (6 SNPs) on Chr16. For body length trait, GWAS showed 48 SNPs, including 6 genome-wide SNPs (*p* < 10^−5^) localized on Chr3, Chr10, Chr13, Chr18 and Chr29, and 42 suggestive SNPs (*p* < 10^−4^) found on Chr1, Chr2, Chr3, Chr5, Chr6, Chr8, Chr9, Chr10, Chr11, Chr12, Chr13, Chr15, Chr16, Chr17, Chr18, Chr20, Chr21, Chr26, Chr27, Chr28 and Chr29. The highest number of SNPs was found on Chr10 (7 SNPs, including 1 genome-wide SNP) and Chr29 (4 SNPs, including 2 genome-wide SNP). For chest perimeter trait, we found 4 genome-wide SNPs (*p* < 10^−5^) located on Chr9, Chr10, Chr18, and Chr19, and 18 suggestive SNPs (*p* < 10^−4^) found on Chr4, Chr5, Chr6, Chr7, Chr9, Chr10, Chr12, Chr13 and Chr17. The largest number of SNPs (3 SNPs, including 1 genome-wide) was detected on Chr9 and Chr10. Chest width revealed 131 SNPs, including 30 genome-wide SNPs (*p* < 10^−5^) found on Chr1, Chr2, Chr3, Chr4, Chr5, Chr7, Chr9, Chr10, Chr12, Chr17, Chr18, Chr20, Chr21, Chr22, Chr24, Chr24, Chr26 and Chr28, and 101 suggestive SNPs (*p* < 10^−4^) localized on all chromosomes except Chr23, Chr24 and Chr25. Part of the SNPs were localized as blocks of several closely spaced SNPs, in particular, 3 SNPs (including 2 genome-wide SNPs) in the region of 59.4–61.7 Mb on Chr4. The highest number of SNPs was found on Chr10 (15 SNPs, including 4 genome-wide SNPs) and Chr9 (13 SNPs, including 3 genome-wide SNPs). GWAS for chest depth showed 8 SNPs, including 1 genome-wide SNP (*p* < 10^−5^) located on Chr18, and 7 suggestive SNPs (*p* < 10^−4^) found on Chr9, Chr13 and Chr17. The highest number of SNPs (3 SNPs) was found on Chr9. Rump width revealed 14 SNPs, including 3 genome-wide SNPs (*p* < 10^−5^) localized on Chr1, Chr2 and Chr3, and 11 suggestive SNPs (*p* < 10^−4^) found on Chr4, Chr8, Ch9, Chr12, Chr14, Chr16, Chr18, Chr20 and Chr25 (Supplementary Materials Table S2). Thus, genome-wide SNPs were identified for all traits studied: body weight (5 SNPs), withers height (7 SNPs), rump height (9 SNPs), body length (6 SNPs), chest perimeter (4 SNPs), chest width (30 SNPs), chest depth (1 SNP) and rump width (3 SNPs) (Table 2).

Among 241 SNPs significantly associated with studied traits, 56 SNPs were common for two or more traits (1 SNP for six traits, 2 SNPs for five traits, 12 SNPs for four traits, 20 SNPs for three traits, and 21 SNPs for two traits), while 185 SNPs were associated with single traits (Supplementary Materials Table S3).

### 3.4. Candidate Genes

The structural annotation revealed 238 genes, which (or part of which) are localized within a window of 0.4 Mb (±0.2 Mb of causal SNPs). The full list of genes is available in the Supplementary Materials, Table S3. Candidate genes for the studied traits were identified on 21 of 29 goat autosomes. The greatest number of candidate genes was found on Chr13 with 33 candidate genes for 5 traits studied. Candidate genes linked to significant SNPs (*p* < 10^−5^) associated with body weight and body conformation traits based on GWAS in Karachai goats at age 8 months are shown in Table 3.

Our results showed that among the total number of identified candidate genes (Supplementary Materials Table S3), the most significant genes associated with body weight at age 8 months are *CRADD*, *HMGA2*, *MSRB3*, *MAX*, *HACL1* and *RAB15*, which are growth factors, including those with high expression in cell growth and development in animal bodies. *APOB*, *PTPRK*, *BCAR1*, *AOAH* and *ASAH1* genes regulate fatty acids metabolism in cells and are involved in various metabolic processes in animal bodies. These genes were noted as being linked to some body conformation traits such as withers height and rump height. The *APOB* gene, associated with intrauterine embryonic development, spermatogenesis, development of the nervous system, cholesterol metabolism, fertilization, and postembryonic development, is noted as being related to withers height, rump height and body length. Furthermore, *WDR70*, *ZBTB24* and *SORCS3* genes were found to be linked to chest width. Moreover, the *KCNG4* gene was showed to be associated with rump height, body length and chest perimeter.

Therefore, *CRADD*, *HMGA2*, *MSRB3*, *MAX*, *HACL1*, *RAB15*, *APOB*, *PTPRK*, *BCAR1*, *AOAH*, *ASAH1*, *WDR70*, *ZBTB24*, *KCNG4* and *SORCS3* genes can be used as molecular markers for growth trait selection in goats, which increases genetic improvement in Russian goat breeds.

Using the DAVID web tool and a list of 238 genes found within the 0.4 Mb regions surrounding the identified SNPs (Supplementary Materials, Table S3), we performed the analysis of functional annotation and enrichment of GO terms (Table 4). We found two significant annotation clusters enriched with GO terms. One of them is related to the cystatin protein subfamily, while the other one is associated with the cell division process.

## 4. Discussion

The study of the goat genome is the main basis for genetic improvement in goat breeding programs. GWAS has been used as a primary strategy to detect QTL (quantitative traits loci) for complex traits [27]. Many genomic regions and candidate genes associated with growth and exterior traits have been identified by means of GWAS in different farm animals [13,14,22,28,29,30], including goats [31].

Our study aimed to identify genomic regions and candidate genes associated with growth and exterior traits in Karachai goats at age 8 months. Karachai (Figure S1) is the aboriginal dual-purpose goat breed, which is bred in high-altitude regions of Caucasus [5]. Phylogenetic studies performed based on Goat SNP BeadChip revealed that the Karachai goats were the most distant among other goat breeds in Russia [32].

The free-range raising of Karachai goats in small herds in the high-altitude areas complicated the phenotypic data collection. In this regard, we managed to obtain reliable body measurements and weights for only 269 goats. This sample size is relatively low for classical GWAS and may be considered as a limitation of our study. However, successful results provided by GWAS in low samples were reported in several previous research works. For example, significant associations with body weight, growth-related and body conformation traits were identified by GWAS in 150 Dazu Black goats [18], in 95 Sudanese goats [28], in 69 Egyptian Barki sheep [33], and in 96 Baluchi sheep [34]. Genome-wide associations with other economic important traits were found in sample sizes of 192 animals [35,36]. In this regard, we believe that it possible to use a sample of 269 animals for our research.

Genome-wide association studies of 269 Karachai goats performed for body weight and seven conformation traits at the age of 8 months using 47,647 SNPs identified genome-wide SNPs (*p* < 10^−5^) for all studied traits, including 5 SNPs for body weight, 7 SNPs for withers height, 9 SNPs for rump height, 6 SNPs for body length, 4 SNPs for chest perimeter, 30 SNPs for chest width, 1 SNP for chest depth, and 5 SNPs for rump width (Table S2). Previous studies identified significant SNPs and obtained genomic regions associated with some of the body conformation traits, which are subjects of our present study. Thus, in Dazu Black goats, 53 significant SNPs were found related to body height, body length, cannon circumference, chest depth, chest width and heart girth [18]. GWAS based on SNP genotypes of 150 Punjab goats and linear body measurements identified two significant SNPs influencing body weight, as well as two, three, four, four and five significant SNPs related to heart girth, height, body length, chest length and pubic bone length, respectively [13]. Several SNPs identified in present work overlapped with genomic regions and QTL associated with body weight and body conformation traits, which were reported in previous studies of goats. Thus, the SNP located on Chr3 (snp6325-scaffold1223-530258, position 107,364,229) was significantly associated with body length (*p* < 10^−5^) in our study and was located near the body length QTL (3: 107,533,545- 107,548,435), which was reported in Sudanese goats [28]. Moreover, three SNPs at Chr5 including snp54059-scaffold822-1742056 (position 8,501,612), snp54060-scaffold822-1781620 (position 8,540,781), and snp15220-scaffold1622-150966 (position 9,865,450) were located nearby the QTL (2–8 cM) associated with growth, which was reported previously in Markhoz goats [37]. A small number of matches between our study and previous results may be due to several reasons. Firstly, discovery of genomic variants, which are involved in the formation of economically important phenotypes in goats, is still lagging behind other livestock species. Moreover, the genetic differences between the goat breeds due to origin and breeding strategies will probably result in revealing various associations. In the present study, the genes related to body weight at 8 months included *CRADD*, *HMGA2*, *CRADD*, *MSRB3*, *MAX*, *HACL1* and *RAB15*. In addition, several genes were associated with body conformations traits in our study, such as *APOB*, *PTPRK*, *BCAR1*, *AOAH*, *ASAH1*, *WDR70*, *ZBTB24*, *SORCS3* and *KCNG*.

In our study, the gene *CRADD* (*CASP2* and *RIPKI* domain containing adaptor with death domain) was related to body weight in Karachai goats at age 8 months. It has been observed that the *CRADD* gene, beside several other genes (*SOCS2* and *PLXNC1*), is localized within the so-called “tall region” on chromosome 10 in mice, mutations in which are related to the appearance of a non-obese tall phenotype in mice [38]. The *CRADD* gene indirectly affects cysteine proteases involved in apoptosis [39]. Therefore, the increase in cell number observed in the tall phenotype appears to be the result of a change in the apoptotic metabolic pathway [40].

A previous study was carried out on the effect of genes localized within the “high growth region” in mice (*CRADD*, *SOCS2* and *PLXNC1*), as well as two closely located genes (*ATP2B1*, *DUSP6*), on the growth, meat and fat quality in pigs. There was shown to be a significant relationship between these genes and phenotypic parameters, including growth and fat deposition in pigs [41].

Our study revealed an association between the gene *HMGA2* (high-mobility group AT-hook 2) and the body weight in Karachai goats at age 8 months, the *HMGA2* gene encodes a small, chromatin-associated protein that belongs to the non-histone chromosomal highly mobile group A of the DNA-binding protein family, this protein can modulate transcription and enhance or inhibit the action of transcriptional enhancers by altering chromatin structure or facilitating the assembly of transcription factor multiprotein complexes [42,43,44].

Several studies have revealed a high level of *HMGA2* expression during embryogenesis; analysis of expression patterns showed the main role of this gene in the determination of growth and development [45,46]. Knockout of the *HMGA2* gene in mice demonstrated the involvement of this gene in diet-induced obesity [47]. Using *CRISPR/Cas9*, a null *HMGA2* allele was generated in mice in which only the coding sequence was specifically disrupted. Loss of one or both *HMGA2* alleles has resulted in a 20% and 60% reduction in body weight, respectively, compared to wild-type littermates, as well as an allometric reduction in skull length, which shows the important role of this gene in mice body weight [48]. Similarity, several previous studies found that the *HMGA2* gene has been shown to be related to growth in humans [49,50]. At the same time, in Duroc pigs, the *HMGA2* gene was identified as one of the candidate genes linked to growth traits [51]. In numerous studies, evidence of a close association between *HMGA2* gene expression and pig weight was also found; the *HMGA2* gene is activated only during early postnatal development and controls the total number of cells in the animal. In particular, the level of its expression is proportional to the animal body weight [52,53]. Thus, the involvement of *HMGA2* in the regulation of prenatal and postnatal growth in various animal and human species was confirmed. Summarizing, we can conclude that all the functions described above are directly or indirectly associated with average daily gains and consequently with an increase in body weight.

In the present study, the gene *MSRB3* (methionine sulfoxide reductase B3) was found to be linked to body weight in Karachai goats at age 8 months. The *MSRB3* gene is one of the most important members of the *MSRB* gene family; it can reduce the catalytic effect of methionine-R-sulfoxide to methionine, particularly as an oxidoreductase [54]. Many previous studies have shown that the *MSRB3* gene can influence the shape and size of ears in pigs and sheep [55,56]. Based on genome-wide association analysis, gene silencing and protein precipitation, the *MSRB3* gene was considered as a candidate gene affecting the growth performance of cattle [57].

More recent studies have shown that indels in the *MSRB3* gene are associated with growth and development indicators (body weight, body length, rump height, chest girth behind the shoulder blades) in four Chinese native cattle breeds [58].

Our research indicates an association between the gene *MAX* (MYC-associated factor X) and body weight in Karachai goats at age 8 months. The protein encoded by the *MAX* gene is a member of the bHLHZ family of transcription factors. It is able to form homodimers and heterodimers with other family members which include MAD, MXI1 and MYC. MYC is an oncoprotein involved in cell proliferation, differentiation and apoptosis. *MAX*, as a partner of MYC, is involved in the control of cell proliferation [59]. The SNP in the *MAX* gene has been shown to be associated with growth in humans [60].

The gene *HACL1* (hydroxyacyl-CoA lyase 1) has been related to body weight; this gene plays an important role in fatty acid oxidation and the fatty acid metabolism process. A previous study on dairy cattle showed that the gene *HACL1* has been associated with two metabolites (-α-ketoglutarate and succinic acid), which were identified in the gene–metabolite interaction network [61]. For the gene *RAB15* (a member of the RAS oncogene family), there is no information on the relationship with animal growth and development in the previous studies.

Our study revealed an association between the genes *APOB*, *PTPRK*, *AOAH* and *ASAH1* and several body conformation traits in Karachai goats at age 8 months. The gene *APOB* (apoliporotein B) has been associated with withers height, rump height and body length. At the same time, the *APOB* gene has been associated with body growth in chickens [62,63]. Furthermore, a previous study carried out on goats observed that the *APOB*/*Hae*III polymorphism is linked to lactose content, lactation length, and somatic cell score, and also, the *APOB*/*Sma*I polymorphism is associated with lactation length, lactose percentage, and total yield of solids non-fat, lactose, protein, fat, and milk [64]. The gene *PTPRK* (protein tyrosine phosphatase receptor type K) gene has been related to chest perimeter. In a previous study, it was observed that the *PTPRK* gene has been associated with marbling in Nelore cattle [65]. At the same time, we observed that the gene *BCAR1* (breast cancer anti-estrogen resistance1) was related to chest width in Karachai goats at age 8 months, several previous studies carried out on cattle and sheep identified *BCAR1* as a candidate regulatory gene of intramuscular fat deposition and fatty acids content [66,67,68].

In the present study, we also observed that the gene *AOAH* (acyloxyacyl hydrolase) was linked to withers height and rump height, and this gene plays an important role in fatty acid metabolism and the lipopolysaccharide catabolism process [69]. At the same time, the gene *ASAH1* (N-acylsphingosine amidohydrolase 1) was associated with rump height and body length, and this gene plays an important role in the fatty acid metabolism process [70].

Most of the candidate genes which we identified as being associated with body conformation traits in Karachai goats at age 8 months play important roles in the metabolism process, which have a direct effect on the size of these traits in animal bodies.

In our research, we observed that the gene *WDR70* (WD repeat domain 70) was associated with chest width in Karachai goats at age 8 months. In a previous study, the *WDR70* gene has been linked to fertility traits in Chinese and Nordic Holsteins [71]. Moreover, we found that the following genes were associated with chest width in our study: *ZBTB24* (Zinc finger and BTB domain containing 24) and *SORCS3* (Sortilin related VPS10 domain containing receptor 3). At the same time, *KCNG4* (potassium voltage-gated channel modifier subfamily G member 4) has been related to rump height, body length, chest perimeter and chest width in our research. In numerous studies, *KCNG4*, *ZBTB24* and *SORCS3* have been related to the immune system and some diseases in human, but there is no information on the relationship with animal body conformation traits in the previous studies. On the other hand, the *SORCS3* gene has been associated with coat color trait in goats [72]. Furthermore, we found an association between the *ADIPOQ* (adiponectin) gene and chest width. The *ADIPOQ* gene plays a role in the skeletal muscle satellite cells’ differentiation into adipocytes and is potentially involved in intramuscular adipogenesis and postnatal muscle growth in goats [73,74,75].

## 5. Conclusions

In the present study, a genome-wide association studies analysis was carried out for body weight and body conformation traits in 269 Karachai goats at age 8 months. The analysis showed that SNPs were identified for all studied traits, including body weight (5 SNPs), withers height (7 SNPs), rump height (9 SNPs), body length (6 SNPs), chest perimeter (4 SNPs), chest width (30 SNPs), chest depth (1 SNPs) and rump width (5 SNPs). We identified the candidate genes related to body weight at age 8 months included *CRADD*, *HMGA2*, *CRADD*, *MSRB3*, *MAX*, *HACL1* and *RAB15*. At the same time, several genes were obtained associated with body conformation traits in our study, which included: *APOB*, *PTPRK*, *BCAR1*, *AOAH*, *ASAH1*, *WDR70*, *ZBTB24*, *SORCS3*, *ADIPOQ*, and *KCNG*. These results will be useful for the development of genetic selection programs aimed at genetic improvement and will increase the productive efficiency of goats.

## Figures and Tables

**Table 1:** Descriptive GLM statistics for body weight and body conformation traits in Karachai goats at age 8 months.

Trait	Max	Min	Mean	Var	Std. Dev	CV
BW	49.5 kg	27.1 kg	36.35 kg	13.1	3.62	9.95
WH	60.4 cm	47.5 cm	52.78 cm	7.3	2.70	5.11
RH	60.5 cm	48.0 cm	53.48 cm	8.1	2.84	5.31
BL	61.5 cm	48.1 cm	54.14 cm	8.4	2.90	5.35
CP	68.9 cm	52.2 cm	60.60 cm	10.0	3.17	5.23
CW	14.0 cm	8.0 cm	10.21 cm	1.5	1.22	11.94
CD	26.4 cm	18.0 cm	21.54 cm	2.7	1.63	7.57
RW	13.9 cm	9.5 cm	11.30 cm	0.6	0.78	6.86

BW: body weight, WH: withers height, RH: rump height, BL: body length, CP: chest perimeter, CW: chest width, CD: chest depth, RW: rump width, Var: variation, Std. Dev: standard deviation, CV: coefficient of variation.

**Table 2:** Quantitative distribution of significant SNPs on chromosomes associated with body weight and body conformation traits in Karachai goats at age 8 months.

Trait	Genome-Wide SNPs (*p* < 10^−5^)		Suggestive SNPs (*p* < 10^−4^)	
	n	Chr	n	Chr
BW	5	5, 6, 10, 16	22	1, 5, 10, 16, 18, 20, 24, 25, 26
WH	7	1, 3, 8, 9, 10, 13, 18	42	2, 3, 4, 5, 6, 7, 8, 9, 10, 11, 13, 15, 16,17, 20, 26, 29
RH	9	1, 3, 8, 9, 10, 13, 18, 26, 29	46	2, 3, 4, 5, 6, 7, 8, 9, 10, 11, 12, 13, 15, 16, 17, 18, 20, 26, 27, 29
BL	6	3, 10, 13, 18, 29	42	1, 2, 3, 5, 6, 8, 9, 10, 11, 12, 13, 15, 16, 17, 18, 20, 21, 26, 27, 28, 29
CP	4	9, 10, 18, 19	18	4, 5, 6, 7, 9, 10, 12, 13, 17
CW	30	1, 2, 3, 4, 5, 7, 9, 10, 12, 17,18, 20, 21, 22, 24, 26, 28	101	1, 2, 3, 4, 5, 6, 7, 8, 9, 10, 11, 12, 13, 14, 15, 16, 17, 18, 19, 20, 21, 22, 26, 27, 28, 29
CD	1	18	7	9, 13, 17
RW	3	1, 2, 3	11	4, 8, 9, 12, 14, 16, 18, 20, 25,

BW: body weight; WH: withers height; RH: rump height; BL: body length; CP: chest perimeter; CW: chest width; CD: chest depth; RW: rump width; Chr: chromosome.

**Table 3:** Closest candidate genes for genome-wide SNPs (*p* < 10^−5^) associated with body weight and body conformation traits based on GWAS in Karachai goats at age 8 months.

Chr	SNP	Position *	Trait	*p*-Value	R^2^	Genes **
1	snp36273-scaffold435-778554	76 791 560	WH	3.66 × 10^−6^	0.069	*CLDN16*, *CLDN1*, *P3H2*
			CW	5.67 × 10^−6^	0.048	
			RH	7.62 × 10^−6^	0.069	
1	snp40557-scaffold519-2601256	80 175 337	CW	7.04 × 10^−6^	0.110	*ST6GAL*, *ADIPOQ*, *RFC4*, *EIF4A2*, *MIR1248*, *KNG1*, *TBCCD1*, *DNAJB11*, *CRYGS*, *FETUB*, *AHSG*, *HRG*
1	snp46677-scaffold65-1704757	105 833 294	RW	7.14 × 10^−6^	0.002	-
2	snp37767-scaffold464-4950066	37 437 223	CW	8.29 × 10^−6^	0.023	-
2	snp40316-scaffold515-741525	48 011 875	RW	2.15 × 10^−6^	0.021	-
3	snp41843-scaffold545-952728	19 599 119	CW	7.17 × 10^−6^	0.016	-
3	snp48395-scaffold687-2186794	39 664 309	RW	7.09 × 10^−6^	0.028	***UBE2U***, *ROR1*
3	snp42136-scaffold55-4190633	57 469 911	CW	9.16 × 10^−6^	0.006	-
3	snp24555-scaffold2498-324452	103 535 122	WH	2.93 × 10^−7^	0.058	*INTS3*, *SLC27A3*, *NPR1*, *CHTOP*, *ILF2*, *SNAPIN*, *NUP210L*, *DENND4B*, *RAB13*, *GATAD2B*, *CREB3L4*, *CRTC2*, *JTB*, *RPS27*
			RH	2.36 × 10^−6^	0.058	
			BL	4.80 × 10^−6^	0.051	
			CW	9.93 × 10^−6^	0.017	
4	snp56760-scaffold9-422861	56 008 260	CW	9.16 × 10^−6^	0.017	-
4	snp31180-scaffold345-2038562	64 439 198	CW	2.69 × 10^−6^	0.016	-
4	snp44315-scaffold603-2039906	104 315 554	CW	6.79 × 10^−6^	0.043	-
5	snp17488-scaffold1809-252855	9 865 450	CW	9.13 × 10^−8^	0.004	-
5	snp38426-scaffold486-2412676	23 345 368	BW	4.83 × 10^−6^	0.067	***CRADD***
6	snp40083-scaffold511-2344051	62 949 329	BW	3.01 × 10^−6^	0.085	-
6	snp40053-scaffold511-1070977	64 228 064	BW	6.44 × 10^−6^	0.065	-
7	snp50806-scaffold735-500181	27 813 856	CW	6.75 × 10^−6^	0.002	-
8	snp12933-scaffold1499-1805970	68 115 674	RH	2.88 × 10^−6^	0.074	-
			WH	2.98 × 10^−6^	0.076	-
9	snp3895-scaffold1122-1069822	23 117 311	CW	2.93 × 10^−6^	0.024	-
9	snp10101-scaffold1358-1351350	27 999 512	CW	1.38 × 10^−6^	0.030	-
9	snp59091-scaffold969-2758702	30 398 000	CW	6.58 × 10^−6^	0.013	-
9	snp29395-scaffold318-901939	53 346 107	CP	4.65 × 10^−6^	0.066	***PTPRK***, *THEMIS*
9	snp3575-scaffold1110-924176	59 047 836	WH	8.69 × 10^−6^	0.071	*EYA4*, *TBPL1*, *TCF21*
10	snp43119-scaffold572-3093664	5 115 781	CP	4.28 × 10^−6^	0.051	-
10	snp1448-scaffold104-1147808	25 854 668	BW	4.56 × 10^−6^	0.049	*MAX*, *RAB15*, *GPX2*
10	snp38108-scaffold475-788703	38 683 034	WH	2.60 × 10^−6^	0.066	-
			RH	3.50 × 10^−6^	0.062	-
			BL	6.24 × 10^−6^	0.069	-
10	snp57312-scaffold912-2276709	53 713 433	CW	2.79 × 10^−7^	0.062	-
10	snp33139-scaffold388-418538	61 359 453	CW	1.67 × 10^−6^	0.001	-
10	snp17716-scaffold184-400952	74 860 568	CW	8.56 × 10^−6^	0.005	-
10	snp16269-scaffold1711-397417	78 944 115	CW	2.87 × 10^−7^	0.018	-
12	snp35954-scaffold431-1932104	37 043 670	CW	2.17 × 10^−7^	0.022	-
13	snp31438-scaffold348-1638233	77 996 024	RH	1.60 × 10^−7^	0.108	*PTPN1*, *RIPOR3*
			WH	7.62 × 10^−7^	0.108	
			BL	2.13 × 10^−6^	0.107	
16	snp8624-scaffold131-2001386	57 408 452	BW	8.21 × 10^−6^	0.047	-
17	snp35564-scaffold428-2794282	38 994 171	CW	5.81 × 10^−6^	0.024	-
17	snp21166-scaffold207-2532534	52 935 461	CW	9.81 × 10^−6^	0.003	-
18	snp6168-scaffold1217-1985930	3 837 951	CW	1.25 × 10^−6^	0.166	*BCAR1*, *LDHD*, *BCNT*, *P97BCNT*
18	snp41877-scaffold546-944746	11 898 552	CD	1.45 × 10^−6^	0.067	*KCNG4*, *NECAB2*, *TAF1C*, *HSDL1*, *MBTPS1*, *DNAAF1*, *ADAD2*, *ATP2C2*, *WFDC1*, *MEAK7*
			BL	1.56 × 10^−6^	0.063	
			WH	1.57 × 10^−6^	0.055	
			RH	2.71 × 10^−6^	0.055	
			CP	5.63 × 10^−6^	0.071	
19	snp32196-scaffold3643-64285	50 562 838	CP	5.91 × 10^−6^	0.076	-
20	snp52283-scaffold776-291202	36 698 503	CW	3.55 × 10^−6^	0.079	*WDR70*, *GDNF*
20	snp46145-scaffold637-12802	46 398 322	CW	4.28 × 10^−6^	0.012	-
21	snp38794-scaffold492-1318578	41 944 739	CW	4.60 × 10^−6^	0.014	-
22	snp55997-scaffold870-361893	58 798 676	CW	8.19 × 10^−6^	0.050	-
24	snp1555-scaffold1042-1068020	33 188 152	CW	6.31 × 10^−6^	0.079	*LAMA3*, *ANKRD29*, *NPC1*
24	snp13566-scaffold1525-519249	61 541 989	CW	6.43 × 10^−6^	0.048	-
26	snp34795-scaffold413-993146	2 502 077	RH	6.66 × 10^−6^	0.040	*GLRX3*
26	snp55577-scaffold861-1367391	26 021 230	CW	6.25 × 10^−6^	0.060	***SORCS3***
28	snp12240-scaffold1457-284782	43 341 968	CW	7.51 × 10^−7^	0.016	-
29	snp19092-scaffold192-2366	16 138 152	BL	6.40 × 10^−6^	0.080	*TENM4*
			RH	6.49 × 10^−6^	0.081	
29	snp14507-scaffold1585-179608	22 176 436	BL	2.65 × 10^−6^	0.034	*ANO5*
			RH	5.99 × 10^−6^	0.039	

* Position of SNP is indicated according ARS1.2 genome assembly; ** Bold type indicates genes within which the identified SNPs are localized.

**Table 4:** Functional annotation and enrichment of gene ontology (GO) terms among the identified genes within the 0.4 Mb regions surrounding the identified SNPs.

Category	GO Term	n	*p*-Value	FE ^1^	FDR ^2^	Genes	
Annotation cluster 1: Enrichment Score: 4.76							
GOTERM_MF_ DIRECT	GO:0004869~cysteine-type endopeptidase inhibitor activity	6	5.60 × 10^−6^	22.39	0.0015	*AHSG*, *FETUB*, *HRG*, *CST7*, *KNG1*	*SPOCK1*
SMART	SM00043:CY	5	2.64 × 10^−5^	27.13	0.0031		
INTERPRO	IPR000010:Proteinase inhibitor I25, cystatin	5	3.49 × 10^−5^	25.67	0.0171		
Annotation cluster 2: Enrichment Score: 1.66							
UP_KW_BIOLOGICAL_PROCESS	KW-0132~Cell division	6	0.0033	22.39	0.0015	*OIP5*, *CHFR*, *DIS3L2*, *AURKA*	*ANKLE2*, *PARD3*
UP_KW_BIOLOGICAL_PROCESS	KW-0131~Cell cycle	7	0.0136	27.13	0.0031		*ANKLE2*, *BRINP2*, *PARD3*
UP_KW_BIOLOGICAL_PROCESS	KW-0498~Mitosis	4	0.0629	25.67	0.0171		
GOTERM_BP_DIRECT	GO:0051301~cell division	5	0.0839	43.72	1		*ANKLE2*

^1^ FE, fold enrichment. ^2^ FDR, false discovery rate.

## Data Availability

The SNP genotypes of 269 Karachai goats used for genome-wide association studies are available in ZENODO repository (doi: 10.5281/zenodo.7053677).

## Supplementary Materials

The following supporting information can be downloaded at: https://www.mdpi.com/article/10.3390/genes13101773/s1, Table S1: Descriptive statistics of genomic inbreeding coefficient distribution in Karachai goats by herds; Table S2: GWAS summary statistics for body weight and body conformation traits in Karachai goats at age 8 months; Table S3: Significant SNPs and candidate genes associated with body weight and body conformation traits in Karachai goats at age 8 months; Figure S1: Karachai goats: (A) 8-month-old female; (B) 8-month-old male; (C) the flock of Karachai goats in its natural habitat; Figure S2: Phenotypic distribution by GLM model for body weight and body conformation traits in Karachai goats at age 8 months; Figure S3: Heatmap of genomic relationship matrix for studied goat populations (herds); Figure S4: Genomic inbreeding coefficient distribution derived from genomic relationship matrix in studied goats of Karachai breed by herds. 1. Darinck; 2. Kyzyl Kala; 3. Mayskiy; 4. Piatigorsky; 5. Storozhevaya; 6. Uchkulan; 7. The whole population of the studied goats.
